# Conditional Lie-Bäcklund Symmetries and Differential Constraints of Radially Symmetric Nonlinear Convection-Diffusion Equations with Source

**DOI:** 10.3390/e22080873

**Published:** 2020-08-08

**Authors:** Lina Ji, Rui Wang

**Affiliations:** Department of Information and Computational Science, He’nan Agricultural University, Zhengzhou 450002, China; rhoda3221@henau.edu.cn

**Keywords:** conditional Lie-Bäcklund symmetry, differential constraint, nonlinear convection-diffusion equation, symmetry reduction, dynamical system

## Abstract

A conditional Lie-Bäcklund symmetry method and differential constraint method are developed to study the radially symmetric nonlinear convection-diffusion equations with source. The equations and the admitted conditional Lie-Bäcklund symmetries (differential constraints) are identified. As a consequence, symmetry reductions to two-dimensional dynamical systems of the resulting equations are derived due to the compatibility of the original equation and the additional differential constraint corresponding to the invariant surface equation of the admitted conditional Lie-Bäcklund symmetry.

## 1. Introduction

The method of conditional Lie-Bäcklund symmetry (CLBS) that was introduced by Fokas and Liu [1], and independently by Zhdanov [2], is the generalization of Lie-Bäcklund symmetry [3] and of conditional (non-classical) symmetry [4,5]. As an application of this method, physically interesting exact solutions, including multi-shock and multi-soliton solutions, are constructed for a large number of nonlinear non-integrable evolution equations in [1,6]. The exact solutions related to invariant subspace for nonlinear equations with quadratic nonlinearity are derived due to linear CLBS in [7]. CLBS is also responsible for the method of invariant subspaces [8], in which the partial differential equation (PDE) is reduced to a system of ordinary differential equations (ODEs). The property of reducibility of evolution equation to several ODEs is in one-to-one correspondence with its CLBS was carried out by Zhdanov in [2]. The CLBSs related to invariant subspace [9], sign-invariant [10,11] and separation of variables [12] are proved to be very powerful to study the classifications and reductions of nonlinear diffusion equations. The reduction theorem for CLBS of evolution system, which generalize the one for scalar case in [2], was presented by Ji and Qu in [13]. The studies about CLBS related to invariant subspace and sign-invariant of nonlinear diffusion system can refer to our work [13,14].

It is stated that the conditional invariance criterion and the compatibility condition are always the same for the evolution equations [14,15]. In fact, the invariant surface condition is right the different constraint (DC) [16] compatible with the considered equation. The method of DC may be too difficult to be practical, since the compatibility condition is too general to be efficient. The effective way is to study CLBSs and DCs in particular cases [9,10,11,12].

Various generalizations of the diffusion equations have been widely studied due to the wide range of applications covering almost every field. An important nonlinear generalization of the diffusion equation is given by

$$\begin{array}{c} u_{t}={\left(u^{k}{\left|u_{x}\right|}^{m-1}u_{x}\right)}_{x}, \end{array}$$

which is due to the nonlinear Fourier law and it has been applied in some situations, including nonlinear heat conduction [17] and nonlinear shear flows of non-Newtonian fluids [18]. The presence of external force and absorbent (source) term will extend the above nonlinear diffusion equation to

$$\begin{array}{c} u_{t}={\left(u^{k}{\left|u_{x}\right|}^{m-1}u_{x}\right)}_{x}+C\left(x,u\right)u_{x}+A\left(x,u\right), \end{array}$$

where C(x,u) and A(x,u) denotes the external force and absorbent rate, respectively. This equation can model numerous scenarios, such as the viscous fluid [19], turbulent diffusion [20], and diffusion in superconductors [21].

A direct multi-dimensional generalization to the nonlinear equation reads as

$$\begin{array}{c} u_{t}=div\left(u^{k}{\left|▽u\right|}^{m-1}▽u\right)+\vec{C}\left(x_{1},\;x_{2},\;\cdots ,\;x_{n},\;u\right)\cdot ▽u+A\left(x_{1},\;x_{2},\;\cdots ,\;x_{n},\;u\right), \end{array}$$

where $\vec{C}\left(x_{1},\;x_{2},\;\cdots ,\;x_{n},\;u\right)$ and $A(x_{1},\;x_{2},\;\cdots ,\;x_{n},\;u)$ are, respectively, the drift and source terms. Lie’s classical symmetry [22,23,24,25,26,27] and conditional symmetry [27,28] has been used to study reductions of (1 + 2)-dimensional and (1 + 3)-dimensional nonlinear diffusion equations, which are right various different particular cases of this multi-dimensional diffusion equation.

The purpose of this manuscript is to study second-order CLBS with the characteristic

(1) $$\begin{array}{c} \sigma =u_{rr}+H\left(u\right)u_{r}^{2}+G\left(r,u\right)u_{r}+F\left(r,u\right) \end{array}$$

of the radially symmetric nonlinear convection-diffusion equation

(2) $$\begin{array}{c} u_{t}=\frac{1}{r^{n-1}}{\left(r^{n-1}u^{k}u_{r}^{m}\right)}_{r}+Q\left(r,u\right)u_{r}+P\left(r,u\right). \end{array}$$

It is noted that H,G,F,P, and *Q* in (1) and (2) are analytic functions of the indicated variables. The studies from applicability point of view about variant special forms of (2) is referred to [29,30,31]. The complete descriptions of the Lie symmetries of the special case of (2) can refer to the recent paper [26].

In [10,11,32], second-order CLBS possessing the form (1) has been proved to be very powerful to study classifications and reductions for all kinds of variant forms of Equation (2). Second-order CLBS of nonlinear diffusion equation can be translated into first-order Hamilton–Jacobi sign-invariant of the considered equation, which is extensively discussed in [10,11,32]. Here, we are mainly concerned with finding all possible CLBSs with the form (1) admitted by Equation (2). As a consequence, the resulting CLBS admitted by Equation (2) will yield the corresponding symmetry reductions due to the compatibility of the original Equation (2) and the invariant surface equation

(3) $$\begin{array}{c} \sigma =u_{rr}+H\left(u\right)u_{r}^{2}+G\left(r,u\right)u_{r}+F\left(r,u\right)=0, \end{array}$$

which is right second-order DC of Equation (2).

The outline of the following paper is listed, as below. We review some basic definitions and notations about the CLBS method and DC method in the next section. Equation (2) admitting CLBSs (1) are identified in Section 3. The consequent reductions of the resulting Equation (2) that are listed in Section 3 are constructed in Section 4. The last section is a summary and discussion about our results.

## 2. General Statement

Let us recall basic facts on CLBS and DC of nonlinear evolution equation.

Consider a nonlinear evolution equation

(4) $$\begin{array}{c} u_{t}=E\left(t,x,u,u_{r},\cdots ,u_{nr}\right) \end{array}$$

with evolutionary vector field

(5) $$\begin{array}{c} V=\sum_{k=0}^{\infty }D_{r}^{k}\eta \left(r,t,u,u_{r},\cdots ,u_{lr}\right)\frac{\partial }{\partial u_{kr}}, \end{array}$$

where we use the following notations

$$\begin{array}{c} D_{r}=\frac{\partial }{\partial r}+\sum_{k=0}^{\infty }u_{(k+1)r}\frac{\partial }{\partial u_{kr}},\;D_{r}^{j+1}=D_{r}\left(D_{r}^{j}\right),\;D_{r}^{0}=1,\;u_{kr}=\frac{\partial^{k}u}{\partial r^{k}}. \end{array}$$

**Definition** **1.** *[3] The evolutionary vector field (5) is said to be a Lie-Bäcklund symmetry of Equation (4) if and only if*

$$\begin{array}{c} V\left(u_{t}-E\right){|}_{L}=0, \end{array}$$

*where L is the set of all differential consequences of (4), i.e.,*

$$\begin{array}{c} D_{r}^{j}D_{t}^{k}\left(u_{t}-E\right)=0,\;j,k=0,1,2,\cdots . \end{array}$$

**Definition** **2.** *[1,2] The evolutionary vector field (5) is said to be a CLBS of Equation (4) if and only if*

(6) $$\begin{array}{c} V\left(u_{t}-E\right){|}_{L\cap M_{r}}=0, \end{array}$$

*where $M_{r}$ denotes the set of all differential consequences of equation η=0 with respect to r, i.e., $D_{r}^{j}\eta =0,$j=0,1,2,⋯.*

A direct computation of (6) will yield

(7) $$\begin{array}{c} D_{t}{\eta |}_{L\cap M_{r}}=0, \end{array}$$

which is nothing but the compatibility condition of the original Equation (4) and the additional DC

(8) $$\begin{array}{c} \eta (r,t,u,u_{r},\cdots ,u_{lr})=0. \end{array}$$

**Definition** **3.** *[33] The DC (8) and the evolution Equation (4) satisfy the compatibility condition if*

(9) $$\begin{array}{c} D_{t}{\eta |}_{L_{r}\cap M_{r}}=0, \end{array}$$

*where $L_{r}$ denotes the set of all differential consequences of (4) with respect to r, i.e., $D_{r}^{j}\left(u_{t}-E\right)=0,$j=0,1,2,⋯.*

## 3. CLBSs (1) of Equation (2)

Because Equation (2) admits of CLBS (1), a direct computation of the conditional invariance criterion (7) gives

$$\begin{array}{cc} D_{t}{\sigma |}_{L\cap M_{r}}\equiv & [D^{\prime \prime \prime }-\left(3m+1\right)HD^{\prime \prime }-3mH^{\prime }D^{\prime }+m\left(3m+2\right)H^{2}D^{\prime }\\ & -mDH^{\prime \prime }-m^{2}\left(m+1\right)DH^{3}+m\left(3m+1\right)DHH^{\prime }]u_{r}^{m+3} \end{array}$$

(10) $$\begin{array}{c} \begin{array}{c} +\{\frac{n-1}{r}\left[D^{\prime \prime }-\left(2m-1\right)HD^{\prime }+m\left(m-1\right)DH^{2}-\left(m-1\right)DH^{\prime }\right]\\ -\left(3m+2\right)GD^{\prime \prime }+m[3\left(2m+1\right)HD^{\prime }G+\left(3m+1\right)DGH^{\prime }\\ -m\left(3m+1\right)DGH^{2}+\left(3m-1\right)DHG_{u}-3D^{\prime }G_{u}-DG_{uu}]\}u_{r}^{m+2}\\ -\{\frac{n-1}{r}\left[\left(m-1\right)DG_{u}+2mGD^{\prime }-2m\left(m-1\right)DHG\right]\\ +\frac{2(n-1)}{r^{2}}\left[D^{\prime }-\left(m-1\right)DH\right]+\left(3m+1\right)D^{\prime }G_{r}+3\left(m+1\right)FD^{\prime \prime }\\ -m[\left(3m+1\right)G^{2}D^{\prime }-m\left(3m-1\right)DHG^{2}+\left(3m-1\right)DHG_{r}\\ +\left(3m-1\right)DGG_{u}+2\left(3m+1\right)HFD^{\prime }+\left(3m+1\right)DFH^{\prime }\\ +3\left(m-1\right)DHF_{u}-\left(3m^{2}-m+2\right)DFH^{2}-3F_{u}D^{\prime }-DF_{uu}\\ -2DG_{ru}]\}u_{r}^{m+1}-\{\frac{n-1}{r}[mDG_{r}-m\left(m-1\right)DG^{2}\\ -2m\left(m-1\right)DHF+\left(m-1\right)DF_{u}+\left(2m+1\right)FD^{\prime }]-\frac{n-1}{r^{2}}D\\ \left[\left(2m-1\right)G+\frac{2}{r}\right]+\left(3m+1\right)D^{\prime }F_{r}+m[2DF_{ru}+DG_{rr}\\ -\left(3m-1\right)DGG_{r}+m\left(m-1\right)DG^{3}-3\left(m-1\right)DHF_{r}\\ -\left(3m-1\right)DFG_{u}-\left(6m+1\right)D^{\prime }GF+2\left(3m^{2}-3m+2\right)DFGH\\ -3\left(m-1\right)DGF_{r}]\}u_{r}^{m}-m\{\frac{n-1}{r}D\left[F_{r}-2\left(m-1\right)FG-\frac{2}{r}F\right]\\ -3\left(m-1\right)DGF_{r}-\left(3m-1\right)DFG_{r}-3\left(m-1\right)DFF_{u}+DF_{rr}\\ +\left(3m^{2}-3m+2\right)DF^{2}H-3mF^{2}D^{\prime }+\left(m-1\right)\left(3m-2\right)DG^{2}F\}u_{r}^{m-1}\\ -m\left(m-1\right)DF\left[\left(3m-4\right)FG-3F_{r}-\frac{n-1}{r}F\right]u_{r}^{m-2}-m\left(m-1\right)\\ \left(m-2\right)DF^{3}u_{r}^{m-3}+\left(-HQ_{u}+Q_{uu}\right)u_{r}^{3}+(H^{\prime }P+HP_{u}+P_{uu}+2Q_{ru}\\ -2GQ_{u})u_{r}^{2}+\left(2P_{ru}+2HP_{r}+PG_{u}+Q_{rr}-3Q_{u}F-QG_{r}-GQ_{r}\right)u_{r}\\ +P_{rr}+PF_{u}-P_{u}F+GP_{r}-QF_{r}-2Q_{r}F, \end{array} \end{array}$$

where the prime denotes the derivative with respect to *u* and the subscripts denotes the partial derivative with respect to the indicated variables. It is noted that $D=u^{k}$ hereafter.

For general *m*, it follows from the expression (10) that the coefficient functions H(u), G(r,u), F(r,u),Q(r,u), and P(r,u) in (1) and (2) satisfy the following over-determined system

$$\begin{array}{c} \begin{array}{c} D^{\prime \prime \prime }-\left(3m+1\right)HD^{\prime \prime }-3mH^{\prime }D^{\prime }+m\left(3m+2\right)H^{2}D^{\prime }-mDH^{\prime \prime }\\ -m^{2}\left(m+1\right)DH^{3}+m\left(3m+1\right)DHH^{\prime }=0,\\ \frac{n-1}{r}\left[D^{\prime \prime }-\left(2m-1\right)HD^{\prime }+m\left(m-1\right)DH^{2}-\left(m-1\right)DH^{\prime }\right]\\ -\left(3m+2\right)GD^{\prime \prime }+m[3\left(2m+1\right)HD^{\prime }G+\left(3m+1\right)DGH^{\prime }\\ -m\left(3m+1\right)DGH^{2}+\left(3m-1\right)DHG_{u}-3D^{\prime }G_{u}-DG_{uu}]=0,\\ \frac{n-1}{r}\left[\left(m-1\right)DG_{u}+2mGD^{\prime }-2m\left(m-1\right)DHG\right]\\ +\frac{2(n-1)}{r^{2}}\left[D^{\prime }-\left(m-1\right)DH\right]+\left(3m+1\right)D^{\prime }G_{r}+3\left(m+1\right)FD^{\prime \prime }\\ -m[\left(3m+1\right)G^{2}D^{\prime }-m\left(3m-1\right)DHG^{2}+\left(3m-1\right)DHG_{r}-2DG_{ru} \end{array} \end{array}$$

(11) $$\begin{array}{c} \begin{array}{c} +\left(3m-1\right)DGG_{u}+2\left(3m+1\right)HFD^{\prime }+\left(3m+1\right)DFH^{\prime }-DF_{uu}\\ +3\left(m-1\right)DHF_{u}-\left(3m^{2}-m+2\right)DFH^{2}-3F_{u}D^{\prime }]=0,\\ \frac{n-1}{r}[mDG_{r}-m\left(m-1\right)DG^{2}-2m\left(m-1\right)DHF+\left(m-1\right)DF_{u}\\ +\left(2m+1\right)FD^{\prime }]-\frac{n-1}{r^{2}}D\left[\left(2m-1\right)G+\frac{2}{r}\right]+\left(3m+1\right)D^{\prime }F_{r}\\ +m[2DF_{ru}+DG_{rr}-\left(3m-1\right)DGG_{r}-3\left(m-1\right)DHF_{r}\\ +m\left(m-1\right)DG^{3}-\left(3m-1\right)DFG_{u}+2\left(3m^{2}-3m+2\right)DFGH\\ -\left(6m+1\right)D^{\prime }GF-3\left(m-1\right)DGF_{r}]=0,\\ \frac{n-1}{r}D\left[F_{r}-2\left(m-1\right)FG-\frac{2}{r}F\right]-3\left(m-1\right)DGF_{r}+DF_{rr}\\ -\left(3m-1\right)DFG_{r}-3\left(m-1\right)DFF_{u}+\left(3m^{2}-3m+2\right)DF^{2}H\\ -3mF^{2}D^{\prime }+\left(m-1\right)\left(3m-2\right)DG^{2}F=0,\\ DF\left[\left(3m-4\right)FG-3F_{r}-\frac{n-1}{r}F\right]=0,\\ -HQ_{u}+Q_{uu}=0,\\ H^{\prime }P+HP_{u}+P_{uu}+2Q_{ru}-2GQ_{u}=0,\\ 2P_{ru}+2HP_{r}+PG_{u}+Q_{rr}-3Q_{u}F-QG_{r}-GQ_{r}=0,\\ P_{rr}+PF_{u}-P_{u}F+GP_{r}-QF_{r}-2Q_{r}F=0,\\ DF^{3}=0. \end{array} \end{array}$$

The last one of (11) implies F=0. As a consequence, the determining system can be reduced to

(12) $$\begin{array}{c} \begin{array}{c} D^{\prime \prime \prime }-\left(3m+1\right)HD^{\prime \prime }-3mH^{\prime }D^{\prime }+m\left(3m+2\right)H^{2}D^{\prime }-mDH^{\prime \prime }\\ -m^{2}\left(m+1\right)DH^{3}+m\left(3m+1\right)DHH^{\prime }=0,\\ \frac{n-1}{r}\left[D^{\prime \prime }-\left(2m-1\right)HD^{\prime }+m\left(m-1\right)DH^{2}-\left(m-1\right)DH^{\prime }\right]\\ -\left(3m+2\right)GD^{\prime \prime }+m[3\left(2m+1\right)HD^{\prime }G+\left(3m+1\right)DGH^{\prime }\\ -m\left(3m+1\right)DGH^{2}+\left(3m-1\right)DHG_{u}-3D^{\prime }G_{u}-DG_{uu}]=0,\\ \frac{2(n-1)}{r^{2}}\left[D^{\prime }-\left(m-1\right)DH\right]+\frac{n-1}{r}[\left(m-1\right)DG_{u}+2mGD^{\prime }\\ -2m\left(m-1\right)DHG]+\left(3m+1\right)D^{\prime }G_{r}-m[\left(3m+1\right)G^{2}D^{\prime }-2DG_{ru}\\ -m\left(3m-1\right)DHG^{2}+\left(3m-1\right)DHG_{r}+\left(3m-1\right)DGG_{u}]=0,\\ -\frac{n-1}{r^{2}}D\left[\left(2m-1\right)G+\frac{2}{r}\right]+\frac{n-1}{r}\left[mDG_{r}-m\left(m-1\right)DG^{2}\right]\\ +m\left[DG_{rr}-\left(3m-1\right)DGG_{r}+m\left(m-1\right)DG^{3}\right]=0,\\ -HQ_{u}+Q_{uu}=0,\\ H^{\prime }P+HP_{u}+P_{uu}+2Q_{ru}-2GQ_{u}=0,\\ 2P_{ru}+2HP_{r}+PG_{u}+Q_{rr}-QG_{r}-GQ_{r}=0,\\ P_{rr}+GP_{r}=0. \end{array} \end{array}$$

Substituting $D=u^{k}$ into the first one of the system (12), we deduce that *H* satisfies the second-order ODE

(13) $$\begin{array}{c} mu^{3}H^{\prime \prime }+m\left[3ku^{2}-\left(3m+1\right)u^{3}H\right]H^{\prime }+m^{2}\left(m+1\right){\left(uH\right)}^{3}\\ -m\left(3m+2\right)k{\left(uH\right)}^{2}+\left(3m+1\right)k\left(k-1\right)uH-k\left(k-1\right)\left(k-2\right)=0. \end{array}$$

It is a nontrivial task to construct the general solution of this nonlinear ODE about H(u). Thus, we cannot list the general solutions of the nonlinear system of PDEs (12). However, even finding particular CLBS (1) can lead to symmetry reductions of the considered Equation (2). In practice, the principle direction of such research is to content oneself with finding CLBS in particular case, and the proper choice is the additional assumption H(u)=h/u. It is noted that *h* is an arbitrary constant. Substituting H(u)=h/u into (13), we get *h* satisfy the algebraic equation

$$\begin{array}{c} (mh-k)(mh-k+1)(mh+h-k+2)=0. \end{array}$$

Therefore,

$$\begin{array}{c} H\left(u\right)=\frac{k}{mu},\;H\left(u\right)=\frac{k-1}{mu},\;H\left(u\right)=\frac{k-2}{(m+1)u} \end{array}$$

are determined. For the case of H(u)=k/m/u, the second one of system (12) will be reduced to a linear ODE about G(r,u). The form of G(r,u) can be determined by solving the second-order linear ODE. When considering the third one and fourth one of system (12), the two undetermined function about *r* in G(r,u) can be made certain. Finally, P(r,u) and Q(r,u) will be presented by solving the last four linear ODE. Here, we omit the detailed procedure for solving system (12) and just summarize the corresponding results in Table 1. The software Maple is used to help us complete the computation procedure.

It is noted from (10) that the terms there may be combined for m=−3,−2,−1,2,3,4,5,6. Consequently, the over-determined systems for these cases are different with (11) for general *m*. However, new results are only found for the case of m=−2,−1,2. Here, we consider the case of m=−2. The over-determined system is listed as

$$\begin{array}{c} \begin{array}{c} -HQ_{u}+Q_{uu}=0,\\ H^{\prime }P+HP_{u}+P_{uu}+2Q_{ru}-2GQ_{u}=0,\\ D^{\prime \prime \prime }+5HD^{\prime \prime }+6H^{\prime }D^{\prime }+8H^{2}D^{\prime }+2DH^{\prime \prime }+4DH^{3}+10DHH^{\prime }+2P_{ru}\\ +2HP_{r}+PG_{u}+Q_{rr}-QG_{r}-GQ_{r}=0,\\ \frac{n-1}{r}\left[D^{\prime \prime }+5HD^{\prime }+6DH^{2}+3DH^{\prime }\right]+4GD^{\prime \prime }+18HD^{\prime }G+10DGH^{\prime }\\ +20DGH^{2}+14DHG_{u}+6D^{\prime }G_{u}+2DG_{uu}+GP_{r}+P_{rr}=0,\\ \frac{-2(n-1)}{r^{2}}\left(D^{\prime }+3DH\right)+\frac{n-1}{r}\left(3DG_{u}+4GD^{\prime }+12DHG\right)+5D^{\prime }G_{r}\\ +10G^{2}D^{\prime }+28DHG^{2}+14DHG_{r}+14DGG_{u}+4DG_{ru}=0,\\ \frac{2(n-1)}{r^{3}}D-\frac{5(n-1)}{r^{2}}DG+\frac{2(n-1)}{r}\left(DG_{r}+3DG^{2}\right)+2DG_{rr}\\ +14DGG_{r}+12DG^{3}=0. \end{array} \end{array}$$

F(r,u)=0 holds for this case. We omit the computational procedure and just list the obtained solutions of this nonlinear system in Table 2. Similarly, we can also present the corresponding results for m=−1 and m=2 in Table 2. It is noted that $h_{1}=-\left(mn+2m-n+2\right)/\left(mn+m-n+1\right)$ and $k_{1}=-\left(m^{2}n+2m^{2}-2mn+m-1\right)/\left(mn+m-n+1\right)$ in Table 1, and a,b,c,d,e,h,p, and *q* in Table 1 and Table 2 are some arbitrary constants. All of the results in Table 1 and Table 2 are also suitable for the special case of n=1.

## 4. Exact Solutions of Equation (2)

The governing Equation (2) and the corresponding CLBS (1) are determined in Section 3. Because the compatibility of the nonlinear diffusion Equation (2) and the invariant surface condition (3) is the key point of CLBS, we first solve the DC (3) to give the form of *u* and then substitute the obtained results into Equation (2) to finally identify *u*. Here, we just list several examples to present the reduction procedure.

**Example** **1.** *Equation*

(14) $$\begin{array}{cc} u_{t}= & \frac{1}{r^{n-1}}{\left(r^{n-1}u^{k}u_{r}^{m}\right)}_{r}\\ & +\left[hr+qr^{\frac{n-1}{m}}-\frac{(m+k)p}{\left(m+1-n\right)}r^{\frac{2m+1-n}{m+1}}+\left(ar^{\frac{n-1}{m}}-\frac{er}{m-n+1}\right)u^{\frac{m+k}{m}}\right]u_{r}\\ & +cu+bu^{-\frac{k}{m}}+\frac{e}{k+m}u^{\frac{2m+k}{m}}+\left(pu+du^{-\frac{k}{m}}\right)r^{\frac{m+1-n}{m}} \end{array}$$

*with k≠−m and m≠n−1 admits of the CLBS*

$$\begin{array}{c} \sigma =u_{rr}+\frac{k}{mu}u_{r}^{2}+\frac{n-1}{mr}u_{r}. \end{array}$$

The corresponding reduction of (14) imply

(15) $$\begin{array}{c} u\left(r,t\right)={\left[\left(m+k\right)\left(\frac{\alpha \left(t\right)}{m+1-n}r^{\frac{m+1-n}{m}}+\frac{\beta \left(t\right)}{m}\right)\right]}^{\frac{m}{m+k}}, \end{array}$$

where α(t) and β(t) satisfy two-dimensional dynamical system

$$\begin{array}{cc} \alpha^{\prime }= & \frac{(m+k)a}{m}\alpha^{2}+\frac{1}{m}\left[(m+k)c+(m+1-n)h\right]\alpha \\ & +\frac{(m+k)(m+1-n)p}{m^{2}}\beta +\frac{(m+k)e}{m^{2}}\alpha \beta +\frac{(m+1-n)d}{m},\\ \beta^{\prime }= & \frac{(m+k)a}{m}\alpha \beta +\frac{(m+k)e}{m^{2}}\beta^{2}+\frac{(m+k)c}{m}\beta +q\alpha +b. \end{array}$$

It is noted that the solutions blow up along the curves $r={\left[-\left((m+1-n)\beta (t)\right)/\left(m\alpha (t)\right)\right]}_{+}^{\frac{m}{m+1-n}}$ for the case of m(m+k)<0 and extinguish along the curves for the case of m(m+k)>0. The ansatz (15) leads to the known time-independent exact solutions of the particular case

$$\begin{array}{c} u_{t}=\frac{1}{r^{n-1}}{\left(r^{n-1}u^{k}u_{r}^{m}\right)}_{r}. \end{array}$$

This type of time-independent solutions has been presented in [26] for the case of n=2 and k=0, where the solutions are constructed due to Lie’s classical symmetry reductions. Another particular case of Equation (14) is listed as

$$\begin{array}{c} u_{t}={\left(u_{r}^{m}\right)}_{r}+auu_{r}, \end{array}$$

which is a generalization of the well-known Burgers equation. The non-trivial solution

$$\begin{array}{c} u\left(r,t\right)=\frac{-r+c_{2}}{at-c_{1}} \end{array}$$

can be derived due to the ansatz (15). Moreover, exact solutions

(16) $$\begin{array}{c} u\left(r,t\right)={\left[c_{2}-\frac{m(m+k)}{\left(m+k\right)\left(m+1-n\right)at+c_{1}}r^{\frac{m+1-n}{m}}\right]}^{\frac{m}{m+k}} \end{array}$$

can be obtained due to the ansatz (15) for the particular case

$$\begin{array}{c} u_{t}=\frac{1}{r^{n-1}}{\left(r^{n-1}u^{k}u_{r}^{m}\right)}_{r}+ar^{\frac{n-1}{m}}u^{\frac{m+k}{m}}u_{r} \end{array}$$

of Equation (14) and the solutions (16) are not obtainable by Lie symmetries.

**Example** **2.** *Equation*

$$\begin{array}{cc} u_{t}= & \frac{1}{r^{n-1}}{\left(r^{n-1}u^{k}u_{r}^{n-1}\right)}_{r}\\ & +\left[h+q\ln r-\frac{(k-1+n)p}{n-1}\ln^{2}r+\left(a-e\ln r\right)u^{\frac{k-1+n}{n-1}}\right]ru_{r}\\ & +cu+bu^{-\frac{k}{n-1}}+\frac{(n-1)e}{k-1+n}u^{\frac{2n-2+k}{n-1}}+\left(pu+du^{-\frac{k}{n-1}}\right)\ln r \end{array}$$

*with k≠1−n admits of the CLBS*

$$\begin{array}{c} \sigma =u_{rr}+\frac{k}{(n-1)u}u_{r}^{2}+\frac{1}{r}u_{r}. \end{array}$$

The corresponding reduction carry out

$$\begin{array}{c} u\left(r,t\right)={\left[\frac{k-1+n}{n-1}\left(\alpha \left(t\right)\ln r+\beta \left(t\right)\right)\right]}^{\frac{n-1}{k-1+n}}, \end{array}$$

where α(t) and β(t) satisfy two-dimensional dynamical system

$$\begin{array}{cc} \alpha^{\prime }= & \frac{(k-1+n)a}{n-1}\alpha^{2}+\frac{(k-1+n)e}{n-1}\alpha \beta +\left[\frac{(k-1+n)c}{n-1}+q\right]\alpha \\ & +\frac{(k-1+n)p}{n-1}\beta +d,\\ \beta^{\prime }= & \frac{(k-1+n)e}{n-1}\beta^{2}+\frac{(k-1+n)a}{n-1}\alpha \beta +\frac{(k-1+n)c}{n-1}\beta +h\alpha +b. \end{array}$$

The solutions blow up along the curves $r=\exp\left[-\beta (t)/\alpha (t)\right]$ for the case of (n−1)(k−1+n)<0 and extinguish along the curves for the case of (n−1)(k−1+n)>0.

**Example** **3.** *Equation*

$$\begin{array}{cc} u_{t}= & \frac{1}{r^{n-1}}{\left(r^{n-1}u^{1-n}u_{r}^{n-1}\right)}_{r}+\left[h+q\ln r-d\ln^{2}r+\left(a+e\ln r\right)\ln u\right]ru_{r}\\ & +[c+b\ln u-e\ln^{2}u+\left(p+d\ln u\right)\ln r]u \end{array}$$

*admits of the CLBS*

$$\begin{array}{c} \sigma =u_{rr}-\frac{1}{u}u_{r}^{2}+\frac{1}{r}u_{r}. \end{array}$$

The corresponding reduction lead to

$$\begin{array}{c} u\left(r,t\right)=r^{\alpha (t)}\exp\left[\beta (t)\right], \end{array}$$

where α(t) and β(t) satisfy two-dimensional dynamical system

$$\begin{array}{c} \alpha^{\prime }=a\alpha^{2}-e\alpha \beta +\left(b+q\right)\alpha +d\beta +p,\\ \beta^{\prime }=-e\beta^{2}+a\alpha \beta +b\beta +h\alpha +c. \end{array}$$

**Example** **4.** *Equation*

$$\begin{array}{cc} u_{t}= & \frac{1}{r^{n-1}}{\left(r^{n-1}u^{-m+1}u_{r}^{m}\right)}_{r}\\ & +\left[hr+qr^{-\frac{1}{m}}-\frac{md}{m+1}r^{\frac{2m+1}{m}}+\left(ar^{-\frac{1}{m}}+\frac{e}{m+1}r\right)\ln u\right]u_{r}\\ & +\left[c+b\ln u-\frac{e}{m}\ln^{2}u+\left(p+d\ln u\right)r^{\frac{m+1}{m}}\right]u \end{array}$$

*with m≠−1 admits of the CLBS*

$$\begin{array}{c} \sigma =u_{rr}-\frac{1}{u}u_{r}^{2}-\frac{1}{mr}u_{r}. \end{array}$$

The corresponding reduction yield

$$\begin{array}{c} u\left(r,t\right)=\exp\left[\frac{m}{m+1}\alpha \left(t\right)r^{\frac{m+1}{m}}+\beta \left(t\right)\right], \end{array}$$

where α(t) and β(t) satisfy two-dimensional dynamical system

$$\begin{array}{cc} \alpha^{\prime }= & \frac{m+1}{m}\alpha^{m+1}-\frac{e}{m}\alpha \beta +a\alpha^{2}+\left[\frac{(m+1)h}{m}+b\right]\alpha \\ & +\frac{(m+1)d}{m}\beta +\frac{(m+1)p}{m},\\ \beta^{\prime }= & n\alpha^{m}-\frac{e}{m}\beta^{2}+a\alpha \beta +b\beta +q\alpha +c. \end{array}$$

**Example** **5.** *Equation*

$$\begin{array}{cc} u_{t}= & \frac{1}{r^{n-1}}{\left(r^{n-1}u^{3}u_{r}^{-2}\right)}_{r}\\ & +\{q\sqrt{r}+sr-4\left(h+1\right)\left[ar^{\frac{3}{2}}-\left(h+1\right)\left(4nh+4n+2h+3\right)r^{2}\right]\\ & +\left(p\sqrt{r}+er\right)u^{h+1}\}u_{r}+\left[c+2a\sqrt{r}-4\left(h+1\right)\left(3nh+3n+2h+3\right)r\right]u\\ & -\frac{e}{2(h+1)}u^{2+h}+\left(d+b\sqrt{r}\right)u^{-h} \end{array}$$

*with h≠−1 admits of the CLBS*

$$\begin{array}{c} \sigma =u_{rr}+\frac{h}{u}u_{r}^{2}+\frac{1}{2r}u_{r}. \end{array}$$

The corresponding reduction bring about

$$\begin{array}{c} u\left(r,t\right)={\left[\left(h+1\right)\left(\alpha \left(t\right)\sqrt{r}+\beta \left(t\right)\right)\right]}^{\frac{1}{h+1}}, \end{array}$$

where α(t) and β(t) satisfy two-dimensional dynamical system

$$\begin{array}{cc} \alpha^{\prime }= & \left(h+1\right)p\alpha^{2}-\frac{(h+1)e}{2}\alpha \beta +\left(h+1\right)a\beta +\left[\left(h+1\right)c+\frac{s}{2}\right]\alpha \\ & +\frac{1}{2}{\left(h+1\right)}^{2}\left(6nh+6n+2h+3\right)\alpha^{-1}\beta^{2}+\frac{b}{2},\\ \beta^{\prime }= & -\frac{(h+1)e}{2}\beta^{2}+\left(h+1\right)p\alpha \beta +\left(h+1\right)c\beta +q\alpha +n{\left(h+1\right)}^{3}\alpha^{-2}\beta^{3}+d. \end{array}$$

The solutions blow up along the curves $r={\left[-\beta (t)/\alpha (t)\right]}^{2}$ for the case of h<−1 and extinguish along the curves for the case of h>−1.

**Example** **6.** *Equation*

$$\begin{array}{cc} u_{t}= & \frac{1}{r^{n-1}}{\left(r^{n-1}u^{2}u_{r}^{-1}\right)}_{r}\\ & +\left\{s+q\ln r-\left(h+1\right)\left[\left(h+1\right)n+a\right]\ln^{2}r+\left(e+p\ln r\right)u^{h+1}\right\}ru_{r}\\ & +cu+du^{-h}-\frac{p}{h+1}u^{h+2}+\left(au+bu^{-h}\right)\ln r \end{array}$$

*with h≠−1 admits of the CLBS*

$$\begin{array}{c} \sigma =u_{rr}+\frac{h}{u}u_{r}^{2}+\frac{1}{r}u_{r}. \end{array}$$

The corresponding reduction suggest

$$\begin{array}{c} u\left(r,t\right)={\left[\left(h+1\right)\left(\alpha \left(t\right)\ln r+\beta \left(t\right)\right)\right]}^{\frac{1}{h+1}}, \end{array}$$

where α(t) and β(t) satisfy two-dimensional dynamical system

$$\begin{array}{cc} \alpha^{\prime }= & \left(h+1\right)e\alpha^{2}-\left(h+1\right)p\alpha \beta +\left[\left(h+1\right)\left(h+2+c\right)+q\right]\alpha \\ & +(h+1)[2(h+1)n+a]\beta +b,\\ \beta^{\prime }= & -\left(h+1\right)p\beta^{2}+\left(h+1\right)e\alpha \beta +{\left(h+1\right)}^{2}n\alpha^{-1}\beta^{2}\\ & +(h+1)(h+2+c)\beta +s\alpha +d. \end{array}$$

The solutions blow up along the curves $r=\exp\left[-\beta (t)/\alpha (t)\right]$ for the case of h<−1 and extinguish along the curves for the case of h>−1.

**Example** **7.** *Equation*

$$\begin{array}{cc} u_{t}= & \frac{1}{r^{n-1}}{\left(r^{n-1}u^{\frac{3-n}{n-1}}u_{r}^{2}\right)}_{r}\\ & +\left[\frac{\left(n-1)(n-7\right)}{4}u^{\frac{2}{n-1}}r^{-2}+\left(n-1\right)\left(ar^{-\frac{1}{2}}+br^{\frac{3}{2}}\right)u^{\frac{1}{n-1}}\right]u_{r}\\ & +\left(cr^{\frac{3}{2}}+dr^{-\frac{1}{2}}\right)u^{\frac{n-2}{n-1}}+\frac{1}{2}{\left(n-1\right)}^{2}\left(a-3br^{2}\right)r^{-\frac{3}{2}}u^{\frac{n}{n-1}} \end{array}$$

*admits of the CLBS*

$$\begin{array}{c} \sigma =u_{rr}+\frac{2-n}{(n-1)u}u_{r}^{2}-\frac{3(n-1)u}{4r^{2}}. \end{array}$$

The corresponding reduction indicate

$$\begin{array}{c} u\left(r,t\right)={\left[\frac{\alpha \left(t\right)-\beta \left(t\right)r^{2}}{2\left(n-1\right)\sqrt{r}}\right]}^{n-1}, \end{array}$$

where α(t) and β(t) satisfy two-dimensional dynamical system

$$\begin{array}{c} \alpha^{\prime }=-\frac{1}{2}\alpha \beta^{2}-a\alpha \beta -b\alpha^{2}+2d,\;\beta^{\prime }=\frac{3}{2}\beta^{3}-a\beta^{2}-b\alpha \beta -2c. \end{array}$$

**Example** **8.** *Equation*

$$\begin{array}{cc} u_{t}= & \frac{1}{r^{n-1}}{\left(r^{n-1}u^{-1}u_{r}^{2}\right)}_{r}\\ & +[s+lr-pr^{2}+\frac{(n-1)e}{3}\ln r+\frac{(n-1)d}{3}\left(\ln r-1\right)r\\ & +\frac{\left(n-1)(n-3\right)}{3r^{2}}+\left(e+dr\right)\ln u]u_{r}-du\ln^{2}u\\ & +\left[q+pr+\frac{(n-1)e}{3r}-\frac{2(n-1)d}{3}\ln r\right]u\ln u\\ & -\frac{{\left(n-1\right)}^{2}d}{9}u\ln^{2}r+\frac{(n-1)p}{3}\left(\ln r-1\right)ru\\ & +\frac{{\left(n-1\right)}^{2}eu\ln r}{9r}+\frac{(n-1)q}{3}u\ln r\\ & +\frac{(n-1)su}{3r}+\frac{{\left(n-1\right)}^{3}u}{27r^{3}}+aru+b \end{array}$$

*admits of the CLBS*

$$\begin{array}{c} \sigma =u_{rr}-\frac{1}{u}u_{r}^{2}-\frac{(n-1)u}{3r^{2}}. \end{array}$$

The corresponding reduction provide

$$\begin{array}{c} u\left(r,t\right)=\exp\left[\alpha (t)r+\beta (t)\right]r^{\frac{1-n}{3}}, \end{array}$$

where α(t) and β(t) satisfy two-dimensional dynamical system

$$\begin{array}{cc} \alpha^{\prime }= & e\alpha^{2}-d\alpha \beta +\left[-\frac{2}{3}\left(n-1\right)d+q+l\right]\alpha +p\beta +a,\\ \beta^{\prime }= & \alpha^{3}+e\alpha \beta -d\beta^{2}+s\alpha +\left[\frac{1}{3}\left(1-n\right)d+q\right]\beta \\ & +\frac{l}{3}\left(1-n\right)+\frac{d}{9}{\left(n-1\right)}^{2}+b. \end{array}$$

## 5. Conclusions

In this paper, second-order CLBS (1) is used to study classifications and symmetry reductions of the radially symmetric nonlinear convection-diffusion equations with source (2). The resulting Equation (2) admitting CLBS (1) is reduced to two-dimensional dynamical system due to the compatibility of the original Equation (2) and the additional DC (3), which is right the invariant surface equation of the admitted CLBS (1). The obtained results generalize the ones for m=1 and n=1 in [10,11] and the special case of Q(r,u)=0 [32]. Exact solutions for certain particular case of Equation (14) are constructed. The obtained solutions include the time-independent cases, the variable separation solutions. The similar discussion can be done for other equations in Examples 2–8. It is shown that some solutions can be derived within the framework of Lie’s classical symmetry, and some of them can be obtained by conditional symmetry. In fact, the second-order CLBS

$$\begin{array}{c} \sigma =u_{rr}+\frac{k}{mu}u_{r}^{2}+\frac{n-1}{mr}u_{r} \end{array}$$

of Equation (2) will yield the first-order invariant surface condition

$$\begin{array}{c} u_{t}-Q\left(r,u\right)u_{r}-P\left(r,u\right)=0, \end{array}$$

which is corresponding to conditional symmetry of Equation (2). The CLBSs of Examples 1, 2, and 3 all degenerate to conditional symmetry cases. In addition, the phenomena of blow up and extinguish are also described due to the resulting solutions.

## Figures and Tables

**Table 1:** CLBS (1) of Equation (2) for general *m* with F(r,u)=0.

No.	Q(r,u)	P(r,u)	H(u)	G(r,u)	*k*	*m*
1	$hr+qr^{\frac{n-1}{m}}-\frac{(m+k)p}{\left(m+1-n\right)}r^{\frac{2m+1-n}{m+1}}$	$cu+bu^{-\frac{k}{m}}+\frac{e}{k+m}u^{\frac{2m+k}{m}}$	$\frac{k}{mu}$	$\frac{n-1}{mr}$	k≠−m	m≠n−1
	$+\left(ar^{\frac{n-1}{m}}-\frac{er}{m-n+1}\right)u^{\frac{m+k}{m}}$	$+\left(pu+du^{-\frac{k}{m}}\right)r^{\frac{m+1-n}{m}}$				
2	$[h+q\ln r-\frac{(k-1+n)p}{n-1}\ln^{2}r$	$cu+bu^{-\frac{k}{n-1}}+\frac{(n-1)e}{k-1+n}u^{\frac{2n-2+k}{n-1}}$	$\frac{k}{(n-1)u}$	$\frac{1}{r}$	k≠1−n	m=n−1
	$+\left(a-e\ln r\right)u^{\frac{k-1+n}{n-1}}]r$	$+\left(pu+du^{-\frac{k}{n-1}}\right)\ln r$				
3	$hr+qr^{\frac{n-1}{m}}-\frac{md}{m+1-n}r^{\frac{2m+1-n}{m}}$	$[c+b\ln u+\frac{e}{m}\ln^{2}u$	$-\frac{1}{u}$	$\frac{n-1}{mr}$	k=−m	m≠n−1
	$+\left(ar^{\frac{n-1}{m}}-\frac{er}{m+1-n}\right)\ln u$	$+\left(p+d\ln u\right)r^{\frac{m+1-n}{m}}]u$				
4	$[h+q\ln r-d\ln^{2}r$	$[c+b\ln u-e\ln^{2}u$	$-\frac{1}{u}$	$\frac{1}{r}$	k=1−n	m=n−1
	+(a+elnr)lnu]r	+(p+dlnu)lnr]u				
5	$hr+qr^{-\frac{1}{m}}-\frac{(m+k-1)p}{m+1}r^{\frac{2m+1}{m}}$	$cu+bu^{\frac{1-k}{m}}-\frac{e}{m+k-1}u^{\frac{2m+k-1}{m}}$	$\frac{k-1}{mu}$	$-\frac{1}{mr}$	k≠−m+1	m≠−1
	$+\left(ar^{-\frac{1}{m}}+\frac{e}{m+1}r\right)u^{\frac{k+m-1}{m}}$	$+\left(pu+du^{\frac{1-k}{m}}\right)r^{\frac{m+1}{m}}$				
6	$hr+qr^{-\frac{1}{m}}-\frac{md}{m+1}r^{\frac{2m+1}{m}}$	$[c+b\ln u-\frac{e}{m}\ln^{2}u$	$-\frac{1}{u}$	$-\frac{1}{mr}$	k=−m+1	m≠−1
	$+\left(ar^{-\frac{1}{m}}+\frac{e}{m+1}r\right)\ln u$	$+\left(p+d\ln u\right)r^{\frac{m+1}{m}}]u$				
7	$hr+qr^{-\frac{2}{m-1}}+\frac{(m-1)p}{mn+m-n+1}r^{\frac{2m}{m-1}}$	$cu+bu^{\frac{mn+2m-n+2}{mn+m-n+1}}$	$\frac{h_{1}}{u}$	$-\frac{2}{(m-1)r}$	$k=k_{1}$	m≠−1
	$+\left(ar^{-\frac{2}{m-1}}+\frac{e}{m+1}r\right)u^{-\frac{m+1}{mn+m-n+1}}$	$+\frac{(mn+m-n+1)e}{\left(m-1)(m+1\right)}u^{\frac{mn-n}{mn+m-n+1}}$			
		$+\left(pu+du^{\frac{mn+2m-n+2}{mn+m-n+1}}\right)r^{\frac{m+1}{m-1}}$				
8	$hr+qr^{\frac{n-1}{m}}+\frac{mp}{m-n+1}r^{\frac{2m-n+1}{m}}$	$cu+bu^{2}-\frac{e}{m}$	$-\frac{2}{u}$	$\frac{n-1}{mr}$	k=−2m	m≠n−1
	$+\left(ar^{\frac{n-1}{m}}-\frac{e}{m+1-n}r\right)u^{-1}$	$+\left(pu+du^{2}\right)r^{\frac{m+1-n}{m}}$				
9	$\left[h+q\ln r+p\ln^{2}r+\left(a-e\ln r\right)u^{-1}\right]r$	$-e+cu+bu^{2}+\left(pu+du^{2}\right)\ln r$	$-\frac{2}{u}$	$\frac{1}{r}$	k=2−2n	m=n−1

**Table 2:** CLBS (1) of Equation (2) for m=−2,−1,2.

No.	Q(r,u)	P(r,u)	H(u)	G(r,u)	F(r,u)	*k*	*m*
1	$q\sqrt{r}+sr+2\left(k-3\right)ar^{\frac{3}{2}}$	$\left(c+a\sqrt{r}\right)u+[d+b\sqrt{r}$	$\frac{2-k}{u}$	$\frac{1}{2r}$	0	k≠3	m=−2
	$+\left(p\sqrt{r}+er\right)u^{3-k}$	−2(k−3)(2kn+k−6n					
		$-4)r]u^{k-2}$					
2	$q\sqrt{r}+sr-4\left(h+1\right)[ar^{\frac{3}{2}}$	$[c+2a\sqrt{r}-4\left(h+1\right)(3nh$	$\frac{h}{u}$	$\frac{1}{2r}$	0	k=3	m=−2
	−(h+1)(4nh+4n+2h	$+3n+2h+3)r]u-\frac{e}{2(h+1)}$
	$+3)r^{2}]+\left(p\sqrt{r}+er\right)u^{h+1}$	$u^{2+h}+\left(d+b\sqrt{r}\right)u^{-h}$					
3	$q\sqrt{r}+sr+2br^{\frac{3}{2}}+(e\sqrt{r}$	$\left(a+b\sqrt{r}\right)u+(d+2c\sqrt{r}$	$-\frac{2}{u}$	$\frac{1}{2r}$	0	k=4	m=−2
	$+pr)u^{-1}$	$-4nr)u^{2}+\frac{p}{2}$					
4	$sr^{\frac{2}{3}}+qr-\frac{9}{2}er^{\frac{4}{3}}$	$cu+du^{\frac{3(3n+2)}{9n+5}}+\left(3n+\frac{5}{3}\right)$	$-\frac{3(3n+2)}{(9n+5)u}$	$\frac{2}{3r}$	0	k=3	m=−2
	$+\frac{9(3n+1)}{{\left(9n+5\right)}^{3}}r^{2}$	$au^{\frac{9n+4}{9n+5}}+\frac{9(3n+1)}{{\left(9n+5\right)}^{2}}ru$
	$+\left(br^{\frac{2}{3}}+ar\right)u^{-\frac{1}{9n+5}}$	$+3\left[pu^{\frac{9n+6}{9n+5}}-\frac{(9n+5)e}{2}u\right]r^{\frac{1}{3}}$					
5	$q\sqrt{r}+sr+\frac{2a}{3(2n+1)}r^{\frac{3}{2}}$	$(c+a\sqrt{r})u$	$-\frac{2(3n+2)}{3(2n+1)u}$	$\frac{1}{2r}$	0	$k=\frac{2(9n+4)}{3(2n+1)}$	m=−2
	$+[\frac{8n}{27{\left(2n+1\right)}^{3}}r^{2}+pr$	$+\left(d+b\sqrt{r}\right)u^{\frac{2(3n+2)}{3(2n+1)}}$
	$+e\sqrt{r}]u^{-\frac{1}{3(2n+1)}}$	$+\frac{9{\left(2n+1\right)}^{3}p+8nr}{6{\left(2n+1\right)}^{2}}u^{\frac{2(3n+1)}{3(2n+1)}}$					
6	$qr^{\frac{2}{3}}+sr-\frac{9}{2}er^{\frac{4}{3}}+(br^{\frac{2}{3}}$	$\frac{3n+1}{3{\left(2n+1\right)}^{2}}ru^{\frac{2(3n+2)}{3(2n+1)}}+du$	$-\frac{2(3n+2)}{3(2n+1)u}$	$\frac{2}{3r}$	0	$k=\frac{2(9n+5)}{3(2n+1)}$	m=−2
	$+ar)u^{-\frac{1}{3(2n+1)}}$	$+\left(2n+1\right)au^{\frac{2(3n+1)}{3(2n+1)}}+bu^{\frac{2(3n+2)}{3(2n+1)}}$
		$+3\left[pu^{\frac{2(3n+2)}{3(2n+1)}}-\frac{3(2n+1)}{2}eu\right]r^{\frac{1}{3}}$					
7	$[s+q\ln r+\left(\frac{k}{2}-1\right)a\ln^{2}r$	$cu+du^{\frac{k}{2}}+\frac{2p}{k-2}u^{2-\frac{k}{2}}-\frac{k}{2}u^{k-1}$	$-\frac{k}{2u}$	$\frac{1}{r}$	0	k≠2	m=−1
	$+\left(e+p\ln r\right)u^{1-\frac{k}{2}}]r$	$+(au+bu^{\frac{k}{2}})\ln r$					
8	{s+qlnr−(h+1)[(h+1)n	$cu+du^{-h}-\frac{p}{h+1}u^{h+2}$	$\frac{h}{u}$	$\frac{1}{r}$	0	k=2	m=−1
	$+a]\ln^{2}r+\left(e+p\ln r\right)u^{h+1}\}r$	$+\left(au+bu^{-h}\right)\ln r$					
9	{s+qlnr+[e+plnr+(k	$cu+du^{3-k}-\frac{2k^{2}-7k+p+6}{k-2}u^{k-1}$	$\frac{k-3}{u}$	$\frac{1}{r}$	0	k≠2	m=−1
	${-2)}^{2}n\ln^{2}r]u^{k-2}\}r$	$+\left[2n\left(2-k\right)u^{k-1}+bu^{3-k}\right]\ln r$					
10	$sr+qr^{1-n}-\frac{(h+1)d}{n}r^{n+1}$	$(c-h-2+dr^{n})u$	$\frac{h}{u}$	$-\frac{n-1}{r}$	0	k=2	m=−1
	$+\left(er+pr^{1-n}\right)u^{h+1}$	$+\left(a+br^{n}\right)u^{-h}-\frac{ne}{h+1}u^{h+2}$
11	$sr+qr^{1-n}-\frac{d}{n}r^{n+1}$	$\left(a-1+br^{n}\right)u+\left(c+dr^{n}\right)u$	$-\frac{1}{u}$	$-\frac{n-1}{r}$	0	k=2	m=−1
	$+\left(er+pr^{1-n}\right)\ln u$	$\ln u-neu\ln^{2}u$					
12	$\frac{\left(n-1)(n-7\right)}{4}u^{\frac{2}{n-1}}r^{-2}+(n$	$\left(cr^{\frac{3}{2}}+dr^{-\frac{1}{2}}\right)u^{\frac{n-2}{n-1}}+\frac{1}{2}(n$	$\frac{2-n}{(n-1)u}$	0	$-\frac{3(n-1)u}{4r^{2}}$	$k=\frac{3-n}{n-1}$	m=2
	$-1)\left(ar^{-\frac{1}{2}}+br^{\frac{3}{2}}\right)u^{\frac{1}{n-1}}$	${-1)}^{2}\left(a-3br^{2}\right)r^{-\frac{3}{2}}u^{\frac{n}{n-1}}$					
13	$s+lr-pr^{2}+\frac{(n-1)e}{3}\ln r$	$-du\ln^{2}u+[q+pr+\frac{(n-1)e}{3r}$	$-\frac{1}{u}$	0	$-\frac{(n-1)u}{3r^{2}}$	k=−1	m=2
	$+\frac{(n-1)d}{3}\left(\ln r-1\right)r$	$-\frac{2(n-1)d}{3}\ln r]u\ln u-\frac{{\left(n-1\right)}^{2}d}{9}$
	$+\frac{\left(n-1)(n-3\right)}{3r^{2}}$	$u\ln^{2}r+\frac{(n-1)p}{3}\left(\ln r-1\right)ru$
	+(e+dr)lnu	$+\frac{{\left(n-1\right)}^{2}eu\ln r}{9r}+\frac{(n-1)q}{3}u\ln r$
		$+\frac{(n-1)su}{3r}+\frac{{\left(n-1\right)}^{3}u}{27r^{3}}+aru+b$					
14	$-\frac{6(3k+9+kn+n)}{{\left(k+1\right)}^{2}r^{2}}u^{k+1}$	$\left(a+br^{3}\right)u^{\frac{1-k}{2}}$	$\frac{k-1}{2u}$	$-\frac{2}{r}$	0	k≠−1	m=2
15	$\frac{(n-9)a}{9}r^{4}u^{\frac{9}{n-9}}+\frac{{\left(n-9\right)}^{2}}{3r^{2}}u^{\frac{18}{n-9}}$	$\left(a+\frac{b}{r^{3}}\right)u^{\frac{n-18}{n-9}}+\frac{{\left(n-9\right)}^{3}}{27r^{3}}u^{\frac{n+9}{n-9}}$	$\frac{18-n}{(n-9)u}$	$-\frac{4}{r}$	0	$k=\frac{27-n}{n-9}$	m=2
16	$\left(\frac{a}{r^{2}}+br\right)\ln u-\frac{9\ln^{2}u}{r^{2}}$	$\left(c+dr^{3}\right)u-3bu\ln^{2}u$	$-\frac{1}{u}$	$-\frac{2}{r}$	0	k=−1	m=2
17	$-\frac{-3n+du+cur^{3}}{r^{2}u^{2}}$	$\left(a+br^{3}\right)u^{2}-3c$	$-\frac{2}{u}$	$-\frac{2}{r}$	0	k=−3	m=2

## Abbreviations

The following abbreviations are used in this manuscript:

CLBS	conditional Lie-Bäcklund symmetry
PDE	partial differential equation
ODEs	ordinary differential equations
DC	differential constraint

## References

[1] Fokas A.S., Liu Q.M. (1994). Nonlinear interaction of traveling waves of nonintegrable equations. Phys. Rev. Lett..

[2] Zhdanov R.Z. (1995). Conditional Lie-Bäcklund symmetry and reductions of equations. J. Phys. A Math. Gen..

[3] Ibragimov N.H. (1989). Transformation Groups Applied to Mathematical Physics.

[4] Bluman G.W., Cheviakov A.F., Anco S.C. (2010). Applications of Symmetry Methods to Partial Differential Equations.

[5] Cherniha R., Serov M., Pliukhin O. (2018). Nonlinear Reaction-Diffusion-Convection Equations: Lie and Conditional Symmetry, Exact Solutions and Their Applications.

[6] Liu Q.M., Fokas A.S. (1996). Exact interaction of solitary waves for certain non-integrable equations. J. Math. Phys..

[7] Liu Q.M. (2001). Exact solutions to nonlinear equations with quadratic nonlinearity. J. Phys. A Math. Gen..

[8] Galaktionov V.A., Svirshchevskii S.R. (2007). Exact Solutions and Invariant Subspaces of Nonlinear Partial Differential Equations in Mechanics and Physics.

[9] Ji L.N., Qu C.Z. (2013). Conditional Lie-Bäcklund symmetries and invariant subspaces to nonlinear diffusion equaitons with convection and source. Stud. Appl. Math..

[10] Qu C.Z., Estévez P.G. (2004). On nonlinear diffusion equations with x-dependent convection and absorption. Nonl. Anal. TMA.

[11] Qu C.Z., Ji L.N., Wang L.Z. (2007). Conditional Lie-Bäcklund symmetries and sign-invarints to quasi-linear diffusion equations. Stud. Appl. Math..

[12] Qu C.Z., Zhang S. L., Liu R.C. (2000). Separation of variables and exact solutions to quasilinear diffusion equations with nonlinear source. Physica D.

[13] Ji L.N., Qu C. Z., Shen S.F. (2014). Conditional Lie-Bäcklund symmetry of evolution system and applications for reaction-diffusion system. Stud. Appl. Math..

[14] Wang J.P., Ji L.N. (2015). Conditional Lie-Bäcklund symmetry, secon-order differential constraints and direct reduction of diffusion systems. J. Math. Anal. Appl..

[15] Olver P. (1994). Direct reduction and differential constraints. Proc. R. Soc. Lond. A.

[16] Sidorov A.F., Shapeev V.P., Yanenko N.N. (1984). Method of Differential Constraints and Its Applications to Gas Dynamics.

[17] Pascal J.P., Pascal H. (1993). On some diffusive waves in non-linear heat conduction. Int. J. Nonlinear Mech..

[18] Pascal J.P., Pascal H. (1995). On some non-linear shear flows of non-Newtonian fluids. Int. J. Nonlinear Mech..

[19] Dieza J.A., Gratton R., Gratton J. (1992). Self-similar solution of the second kind for a convergent viscous grabity currrent. Phys. Fluids A.

[20] Grarilov V.P., Klepikova N.V., Rodean H.C. (1995). Trial of nonlinear diffusion equation as a model of turbulent diffusion. Atmos. Environ..

[21] Vinokur V.M., Feigel’man M.V., Geshkenbein V.B. (1991). Exact solution for flux creep with logarithmic U(j) dependence: Self-organized critical state in high-Tc superconductors. Phys. Rev. Lett..

[22] Bokhari A.H., AI Deweik A.Y., Kara A.H., Zaman F.D. (2010). A symmetry analysis of some classes of evolutionary nonlinear (2+1)-diffusion equations with variable diffusivity. Nonlinear Dyn..

[23] Edwards M.P., Broadbridge P. (1994). Exact transient solutions to nonlinear diffusion-convection equations in higher dimensions. J. Phys. A Math. Gen..

[24] Cherniha R., Kovalenko S. (2011). Lie symmetries and reductions of multi-dimensional boundary value problems of the Stefan type. J. Phys. A Math..

[25] Cherniha R., Serov M., Prystavks Y. (2020). A complete Lie symmetry classification of a class of (1+2)-dimensional reaction-diffusion-convection equations. Commun. Nonlinear Sci. Numer. Simul..

[26] Cherniha R., King J.R., Kovalenko S. (2016). Lie symmetry properties of nonlinear reaction-diffusion equations with gradient-dependent diffusivity. Commun. Nonl. Sci. Numer. Simul..

[27] Cherniha R., King J.R. (2015). Lie and conditional symmetries of a class of nonlinear (1+2)-dimensional boundary value problems. Symmetry.

[28] Broadbridge P., Daly E., Goard J.M. (2017). Exact solutions of the Richards equation with nonlinear pant-root extraction. Water Resour. Res..

[29] Barenblatt G.I. (1952). On self-similar motions of compressible fluid in a porous medium. Prikl. Math. Mekh..

[30] Perona P., Malik J. (1990). Scale-space and edge detection using anisotropic diffusion. IEEE Trans. Pattern Anal. Mach. Intell..

[31] Pascal J.P. (1992). Similarity solutions for axisymmetric plane radial power law fluid flows through a porous meidum. Comput. Math. Appl..

[32] Ji L.N., Feng W. (2018). Second-order conditional Lie-Bäcklund symmetries and differential constraints of nonlinear reaction-diffuison equations with gradient-dependent diffusivity. Symmetry.

[33] Andreev V.K., Kaptsov O.V., Pukhnachev V. V., Rodionov A.A. (1998). Applications of Group-Theoretic Methods in Hydrodynamics.

